# Changes in the sleeping habits of Japanese university students during the COVID-19 pandemic: a 3-year follow-up study

**DOI:** 10.1186/s13030-022-00257-y

**Published:** 2023-04-04

**Authors:** Reiko Hori, Eiji Shibata, Iwao Okajima, Masahiro Matsunaga, Tomohiro Umemura, Akihiko Narisada, Kohta Suzuki

**Affiliations:** 1 Department of Health & Psychosocial Medicine, Aichi Medical University School of Medicine, 1-1 Yazakokarimata, Nagakute, Aichi 480-1195 Japan; 2 Yokkaichi Nursing and Medical Care University, 1200 Kayoucho, Yokkaichi, Mie 512-8045 Japan; 3 Institute for Occupational Health Science, Aichi Medical University, Nagakute, Aichi 480-1195 Japan

**Keywords:** Sleep duration, Delayed sleep phase, University student, COVID-19 pandemic, Remote teaching, Multilevel analysis

## Abstract

**Background:**

The coronavirus disease 2019 (COVID-19) pandemic has greatly changed our daily life. Owing to the imposed restrictions, many educational facilities have introduced remote teaching. This study aims to clarify the association between remote teaching and Japanese university students' sleeping habits.

**Methods:**

The participants were medical students at Aichi Medical University. We used data from an ongoing longitudinal sleeping habits survey. For the participants who enrolled in the university during 2018–2020, multilevel analyses of sleep duration during weekdays and weekends across 3 years were conducted, adjusting for sex, grade, place of stay, sleep problems and lifestyle habits.

**Results:**

Among the students enrolled in the university, the data of 677 in 2018, 657 in 2019, and 398 in 2020 was available for analysis. The mean sleep duration during weekdays (in minutes) was 407.6 ± 60.3 in 2018, 406.9 ± 63.0 in 2019, and 417.3 ± 80.9 in 2020. The mean sleep duration during weekends (in minutes) was 494.5 ± 82.5 in 2018, 488.3 ± 87.9 in 2019, and 462.3 ± 96.4 in 2020. Multilevel analysis conducted for the 684 participants who enrolled during 2018–2020 showed that sleep duration during weekdays was associated with the place of stay and survey year. Moreover, students reported significantly longer sleep duration during weekdays in 2020 than in 2019, but no significant difference in sleep duration was found between 2018 and 2019. The other multilevel analysis found sleep duration during weekends to be associated with the survey year, sex and always doing something before going to bed. Sleep duration during weekends was shorter in 2020 than in 2019 and longer for male students and students who always do something before going to bed. Ten students were reported to have a delayed sleep phase in 2020.

**Conclusions:**

Students' sleep duration increased during weekdays and decreased during weekends in 2020. This difference could be explained by the COVID-19 pandemic and the introduction of remote teaching.

## Background

The coronavirus disease 2019 (COVID-19) pandemic has greatly affected various aspects of our daily life. In Japan, no so-called "urban lockdown" with a penalty was imposed; however, certain directives, such as avoiding unnecessary outings and working remotely from home, were encouraged [1]. Students were also requested to avoid going to school and to engage in self-directed learning early in the pandemic, with a subsequent transition to remote classes or practical assignments in a step-by-step manner.

Sleeping habits, including sleep duration, sleep loss, and sleep quality are known to be related to students' academic achievement [2–5], psychological health [6–8], and physical health [7, 9, 10]. Using the Pittsburgh Sleep Quality Index, a recent meta-analysis [11] reported that the pooled prevalence of poor sleep quality was 52.7% among medical students. However, to our knowledge, there has been no report regarding the sleeping habits of Japanese medical students. We have met young people with school absenteeism and some sleep–wake problems in clinical practice and at a university site. Therefore, we conducted an anonymous pilot study on sleeping habits for our university students. It showed some differences in sleep duration based on sex, grade, and living place, and some students had various sleep–wake problems [12]. We have been conducting an annual survey to collect data on university students' sleeping habits since 2018 to better understand their everyday lives and to analyze their current sleep patterns.

Some reports [13–16] on sleeping habits during the COVID-19 pandemic have been published. A worldwide internet-based Global Chrono Corona Survey that queried sleep–wake times before and during COVID-19 social restriction reported that the individual sleep duration during weekdays increased and sleep duration during work-free days decreased [13]. Moreover, it reported the individual mid-point of sleep delayed in social restriction. However, not all participants answered at these two time points of the survey—that is, before and during the COVID-19 pandemic. Another online survey on sleep, conducted among the general public in Switzerland, Austria, and Germany, showed that the difference between mid-sleep on free days and workdays and the difference in sleep duration between free days and workdays were reduced. Further, sleep duration was increased during the lockdown [14]. A study on the sleep of 139 university students in the U.S. who were taking the same classes remotely during the stay-at-home phase of the pandemic reported an increase in the average sleep duration for both weekdays and weekends during the stay-at-home phase compared to before the pandemic [15]. An observational study on 207 nursing students in Spain, monitored in February and April 2020, showed that they spent more time in bed [16]. Although these studies differ in terms of the surveillance, participants, countries, and measurement methods, they were conducted in countries with strict lockdowns and compared data across a few months. In contrast, in Japan, the state of emergency for COVID-19 was declared on April 7 and lifted on May 25; subsequently, the regulations were eased in stages. However, the general public was directed to work or study remotely in July 2020, and since then, many universities have been conducting remote lectures in conformity with the request. Given this context, we assessed the students' sleep duration and phase during weekdays and weekends from 2018 to 2020. This study was done to determine if medical students' sleeping habits have changed during the COVID-19 pandemic.

## Methods

### Survey design and participants

The present survey was conducted as a part of a prospective survey on sleep behavior, sleep problems, and school life among the students of Aichi Medical University. The study participants were all medical students at Aichi Medical University, which had 707 students enrolled in 2018, 709 in 2019 and 718 in 2020. In 2018 and 2019, a beneficial sleeping habits survey questionnaire was distributed to each participant and collected during their annual physical examination in April. The physical examination was conducted based on the School Health and Safety Act of Japan [17]. The participants were informed of this study's purpose and methods, and were asked to consent to participation by ticking a box in the survey. The consent of the participants who failed to tick the box in the questionnaire was confirmed later by email. In 2020, the survey was conducted online in July because the students could not visit the university campus amid the COVID-19 pandemic.

Usually, the students from the first to the fourth year attend five or six classes a day, starting at 8:50 in the morning. Conversely, the fifth- and sixth-year students participate in clinical practice in the university hospital or community-based hospitals from the morning as per each hospital's schedule. In July 2020, a quarter of the students in the first grade went to school in rotation, and the remaining first-year students and the second- and third-year students were instructed to take remote classes. The students reported living at their own or their parents' homes. The schedule for the remote classes was the same as usual. Because the fifth- and sixth-year students could not go to community-based hospitals, they attended clinical practice at the university hospital, thereby reducing the time and sharing the opportunity.

### Measures

A few second-year students created the questionnaire used in this study as part of a practical assignment for their curriculum in 2016—it was adapted from a questionnaire developed by Shirakawa [18]. The questions were designed to collect information about sleeping habits and living situations, including the following: the hours at which one goes to bed and wakes up during weekdays and weekends; the time spent lying awake in bed before falling asleep and after waking up; how comfortable one feels with the sleep duration; frequency of naps; a self-assessment of one's sleeping depth; one's ease in waking up; what one does before going to bed; the time spent watching TV or using a mobile phone; how one gets up; how often one wakes up early in the morning; arousal frequency during sleep; frequency of going to the bathroom during sleep time; if there is a need to use hypnotics or minor tranquilizers to promote sleep; how frequently sleep paralysis occurs; how frequently one dreams, snores, experiences sleep apnea; if one performs any extracurricular activity, has a part-time job; whether one lives alone or with family; and if one smokes or drinks. The Aichi Medical University Hospital Ethics Board approved this study's design (approval number: 2018-M005, 2020-M015).

### Statistical Analysis

Data were analyzed for 677 students in 2018, 657 in 2019, and 398 in 2020 who responded to the questionnaire. Among them, 199 observations for weekdays and 181 for weekends were treated as missing data. They were as follows: (A) the bedtime or wake-up time was absent (12 observations for weekdays and 21 for weekends in 2018; 12 observations for weekdays and 18 for weekends in 2019), (B) the sleep duration was less than 240 min (7 observations for weekdays and 6 for weekends in 2018; 5 observations for weekdays and 4 for weekends in 2019; 6 observations for weekdays and 4 for weekends in 2020), or (C) it was more than 900 min, and their bedtime and wakeup time were (C-1) between 6:00 and 10:00 and 23:00 and 4:00 (30 observations for weekdays and 16 for weekends in 2018; 45 observations for weekdays and 26 for weekends in 2019;2 observations for weekdays and 2 for weekends in 2020), (C-2) between 10:00 and 15:00 and 5:00 and 12:00 (2 observations for weekends in 2018; 66 observations for weekdays and 39 for weekends in 2019;9 observations for weekdays and 9 for weekends in 2020), (C-3) between 22:00 and 4:00 and 18:00 and 0:00 (1 observation for weekdays and 23 for weekends in 2018; 2 observations for weekdays and 18 for weekends in 2019;1 observation for weekdays in 2020), or another three data (between 8:30 and 8:15 on weekdays in 2020, between 22:00 and 14:00 on weekends in 2019, and between 7:45 and 6:45 on weekends in 2020), respectively. These data were considered bedtime and wake-up time transposed, mixed up 12-h and 24-h notations, or some sort of mistake. For the multilevel analyses, 684 participants who enrolled in the university during 2018–2020 were included. Among the responses collected, 173 observations for weekdays and 161 for weekends were treated as missing data. Such observations included the following: (A) the bedtime or wake-up time was absent (12 observations for weekdays and 21 for weekends in 2018; 6 observations for weekdays and 12 for weekends in 2019); (B) the sleep duration was less than 240 min (7 observations for weekdays and 6 for weekends in 2018; 3 observations for weekdays and 2 for weekends in 2019; 3 observations for weekdays and 3 for weekends in 2020); or (C) it was more than 900 min, and their bedtime and wakeup time were (C-1) between 6:00 and 10:00 and 23:00 and 4:00 (30 observations for weekdays and 16 for weekends in 2018; 40 observations for weekdays and 24 for weekends in 2019;2 observations for weekdays and 2 for weekends in 2020); (C-2) between 10:00 and 15:00 and 5:00 and 12:00 (2 observations for weekends in 2018;60 observations for weekdays and 29 for weekends in 2019;8 observations for weekdays and 7 for weekends in 2020); or (C-3) between 22:00 and 4:00 and 18:00 and 0:00 (1 observation for weekdays and 23 for weekends in 2018;1 observations for weekdays and 14 for weekends in 2019).

All analyses were performed using SAS 9.4 (SAS Institute Inc., Cary, NC, USA). The mean sleep duration during weekdays and weekends was compared using a paired t-test. Multilevel analyses were performed to determine if there was any difference among each sleep duration data for weekdays and weekends across the 3 years, using the SAS PROC MIXED procedure. We did not exclude participants for whom bedtime or wake-up data were missing because SAS PROC MIXED automatically handles missing data using restricted maximum likelihood.

We used the following model to explore the differences in the slopes for each interval of 3 years.

$$Sleep\ duration_{iy}=\beta\mathit{1}+ \beta\mathit{2} \times Gender_{i} + \beta\mathit{3} \times Grade_{iy} + \beta\mathit{4} \times Place\ of\ stay_{iy} + \beta\mathit{5} \times\ Smoking_{iy} + \beta\mathit{6} \times Drinking_{iy} + \beta\mathit{7} \times Extracurricular\ activities_{iy} + \beta\mathit{8}\times Part\text{-}time\ job_{iy} + \beta\mathit{9} \times Doing\ something\ always\ before\ going\ to \ bed_{iy} + \beta\mathit{10}\times Taking\;any\;hypnotics\;or\;minor\;tranquilizer_{iy}+\beta\mathit{11}\times Apnea_{iy}+\beta\mathit{12}\times Arousal\;during\;sleep_{iy}+e_{iy}$$

where *i* represents the individual, *y* represents year, *ꞵ1-12* represents parameters, and *e* is the error term.

The level of statistical significance was set at 5% (*p* < 0.05).

## Results

In 2018, the data of 677 students who answered the questionnaire was available for analysis (response rate was 95.8%). In 2019, 660 students answered the questionnaire, and the data of 657 was available: one student did not wish to be included and two students wrote the same identification number (response rate 92.7%). In 2020, the data of 398 students who answered questionnaire was available for analysis (response rate 55.4%). Their basic data is presented in Table 1.

**Table 1:** Participant characteristics, by year

Variable		2018 (*n* = 677)		2019 (*n* = 657)		2020 (*n* = 398)	
Age		22.0 ± 2.5	650^a^	22.0 ± 2.5	620^a^	21.9 ± 2.6	389^a^
Sex (women)	n, (%)	288 (44.8%)	643^a^	285 (43.9%)	650^a^	167 (42.5%)	393^a^
Student grade			643^a^		650^a^		398^a^
First year	n, (%)	115 (17.9%)		122 (18.8%)		104 (26.1%)	
Second year	n, (%)	114 (17.7%)		115 (17.7%)		74 (18.6%)	
Third year	n, (%)	99 (15.4%)		112 (17.2%)		69 (17.3%)	
Fourth year	n, (%)	94 (14.6%)		99 (15.2%)		60 (15.1%)	
Fifth year	n, (%)	107 (16.6%)		100 (15.3%)		46 (11.6%)	
Sixth year	n, (%)	114 (17.7%)		102 (15.7%)		45 (11.3%)	
Living alone	n, (%)	438 (67.5%)	649^a^	436 (67.7%)	644^a^	189 (48.1%)	393^a^
Takes part in extracurricular activity	n, (%)	446 (67.2%)	664^a^	433 (66.8%)	648^a^	251 (64.0%)	392^a^
Has a part time job	n, (%)	171 (25.8%)	664^a^	178 (27.5%)	648^a^	105 (26.4%)	398^a^
Current smoker	n, (%)	19 (2.9%)	663^a^	21 (3.3%)	646^a^	11 (2.8%)	393^a^
Current drinker	n, (%)	421 (63.4%)	664^a^	418 (35.3%)	646^a^	242 (61.4%)	394^a^

^a^number of valid respondents

The mean sleep duration (in minutes) during weekdays was 407.6 ± 60.3 in 2018, 406.9 ± 63.0 in 2019, and 417.3 ± 80.9 in 2020 (Table 2). Based on the explanatory variables regarding basal characteristics, behaviors, and sleep–wake problems, the multilevel analysis of sleep duration during weekdays through the 3 years with a model that used 1,184 of the 1,536 data sets revealed that sleep duration was related to the grade, place of stay, and survey year (Table 3). Moreover, sleep duration during weekdays in 2020 was significantly longer than in 2019, but there was no significant difference between 2018 and 2019. It was longer for those living alone than for those staying at their family home.

**Table 2:** Mean sleep duration during weekdays and weekends

Year		Mean sleep duration during weekdays (min.)	Mean sleep duration during weekends (min.)	*P*-value
2018	Total	407.6 ± 60.3	494.5 ± 82.6	< 0.0001
	Men	413.4 ± 63.1	485.8 ± 83.4	< 0.0001
	Women	400.1 ± 55.7	505.5 ± 80.1	< 0.0001
2019	Total	406.9 ± 63.0	488.3 ± 87.9	< 0.0001
	Men	408.7 ± 59.2	481.1 ± 85.3	< 0.0001
	Women	404.6 ± 67.8	497.8 ± 90.7	< 0.0001
2020	Total	417.3 ± 80.9	462.3 ± 96.4	< 0.0001
	Men	418.8 ± 86.0	456.6 ± 102.7	< 0.0001
	Women	415.2 ± 74.0	470.1 ± 87.0	< 0.0001

**Table 3:** Multilevel analysis of sleep duration during weekdays in 2018–2020

	Estimate	SE	95% confidence limits		*P*-value
			Lower	Upper	
Men	2.1	4.4	-6.6	10.7	0.64
Women	reference				
2018	-0.9	3.7	-8.1	6.3	0.80
2019	reference				
2020	18.4	5.0	8.6	28.2	0.0003
Family home	reference				
Lodgings	23.3	4.4	14.6	32.1	< .0001
First year	reference				
Second year	14.4	7.7	-0.7	29.4	0.06
Third year	11.6	8.0	-4.1	27.3	0.15
Fourth year	21.2	8.3	4.9	37.5	0.1
Fifth year	14.3	8.3	-2.0	30.5	0.08
Sixth year	20.2	8.3	4.0	36.4	0.01
Always do something before going to bed					
Yes	-0.08	5.6	-11.3	11.1	0.98
no	reference				
Take hypnotics or minor tranquilizer					
Yes	-10.9	12.6	-38.1	16.4	0.41
no	reference				
Sleep apnea					
Yes	1.6	5.0	-8.4	11.6	0.75
no	reference				
Arousal during sleep					
Yes	9.1	5.8	-2.5	20.7	0.12
no	reference				
Extracurricular activities					
Yes	-1.8	4.6	-10.8	7.3	0.70
No	reference				
Part-time job					
Yes	0	4.3	-8.6	8.5	0.99
No	reference				
Smoking					
Yes	10.2	11.7	-17.6	38.0	0.41
No	reference				
Drinking					
Yes	0.2	4.2	-8.1	8.5	0.97
No	reference				

The mean sleep duration (in minutes) during weekends was 494.5 ± 82.6 in 2018, 488.3 ± 87.9 in 2019, and 462.3 ± 96.4 in 2020 (Table 2). The multilevel analysis of sleep duration during weekends through the 3 years, with a model used 1,181 of the 1,536 data sets showed that sleep duration during weekends was related to sex, grade, doing something always before going to bed and survey year (Table 4). Sleep duration during weekends in 2020 was shorter than in 2019, although there was no significant difference between 2018 and 2019. Additionally, sleep duration during weekends was shorter for male students than for female students, and longer among students who consistently do something before going to bed.

**Table 4:** Multilevel analysis of sleep duration during weekends in 2018–2020

	Estimate	SE	95% confidence limits		*P*-value
			Lower	Upper	
Men	-24.4	6.4	-37.1	-11.8	0.0002
Women	reference				
2018	0.3	5.3	-10.1	10.8	0.95
2019	reference				
2020	-25.1	7.2	-39.2	-11.0	0.0005
Family home	reference				
Lodgings	5.0	6.4	-7.9	18.0	0.44
First year	reference				
Second year	24.2	11.3	2.0	46.4	0.03
Third year	26.8	11.8	3.6	50.0	0.02
Fourth year	24.7	12.3	0.7	48.8	0.04
Fifth year	3.9	12.3	-20.2	28.2	0.75
Sixth year	-0.8	12.2	-24.8	23.2	0.95
Always do something before going to bed					
Yes	17.8	8.1	1.6	34.0	0.03
no	reference				
Take hypnotics or minor tranquilizer					
Yes	-9.9	18.2	-49.5	29.7	0.60
no	reference				
Sleep apnea					
Yes	-3.5	7.3	-18.1	11.0	0.63
no	reference				
Arousal during sleep					
Yes	13.0	8.3	-3.6	29.5	0.12
no	reference				
Extracurricular activities					
Yes	-10.8	6.7	-24.1	2.4	0.11
No	reference				
Part-time job					
Yes	2.2	6.3	-10.4	14.8	0.73
No	reference				
Smoking					
Yes	0.3	16.4	-38.6	39.2	0.98
No	reference				
Drinking					
Yes	7.3	6.1	-4.9	19.4	0.24
No	reference				

A total of 10 students went to sleep between 3:00 and 6:00 and got up after 9:00 during weekdays in 2020, compared to two in 2018 and three in 2019. Conversely, 35 students in 2018, 31 students in 2019, and 44 students in 2020 went to sleep between 3:00 and 6:00 and woke up after 9:00 only during weekends.

## Discussion

The annual study on student health and sleeping habits from 2018 to 2020 showed that sleep duration during weekdays increased. In contrast, sleep duration during weekends decreased, in the context of the COVID-19 pandemic in 2020. It also showed that 10 students went to bed early in the morning and woke up late in 2020.

The students' sleep duration during weekdays was shorter than during weekends for each year. This was consistent with the 2015 NHK Japanese Time Use Survey [19], which showed that the mean sleep duration was 7 h and 15 min during weekdays; 7 h and 42 min on Saturday; and 8 h and 3 min on Sunday. It also showed that the mean sleep duration during weekdays was 7 h and 27 min for men in their 20 s and 7 h and 18 min for women in their 20 s; 7 h and 43 min for men in their 20 s and 8 h and 6 min for women in their 20 s on Saturday; and 8 h and 25 min for men in their 20 s and 8 h 27 min for women in their 20 s on Sunday. Although the participants of this study may have slept a shorter duration than responders of the NHK survey in their 20 s did, the difference in sex may denote the same tendency.

Although the sleep duration on weekdays and weekends did not differ between 2018 and 2019, in 2020, the sleep duration increased during weekdays and decreased during weekends. It means the gap between the sleep duration during weekdays and weekends decreased, which is one of indices of sleep deprivation. Furthermore, in 2020, 10 out of 393 students' sleep phase was delayed, while two out of 644 students in 2018 and three out of 649 students in 2019 had a delayed sleep phase. A web-based survey for young adults in Japan [20] showed that time in bed per night and total sleep time in weekdays was longer during the pandemic than before the pandemic, with the same remaining unchanged in weekends. This differing result of sleep time in weekends may be due to the differences in the participants. Our study involved the students of a single medical university, while the aforementioned study recruited participants via an online marketing research company. We posited that the introduction of remote classes during the COVID-19 pandemic resulted in these changes. Remote classes reduce the commute time of students, allowing them to sleep longer and overcome sleep deprivation. However, these classes may have caused a delay in the sleep phase of some students. Instead of having a fixed time, they could take the classes whenever they choose to. Some studies on adolescents reported a later bedtime on weekend nights compared to school nights [21]. Thus, several students may have slept later on weekdays without the usual tight schedule, similar to weekends.

Furthermore, students who always do something before going to bed slept longer during weekends, but not during weekdays. A total of 95% of them used a smartphone or watched TV before going to bed. Because the students must go to school during weekdays, they may get up even if they do not have enough sleep, which might be why they sleep longer during weekends to compensate for the sleep debt accumulated on weekdays [22].

The students' sleep duration on weekdays was not associated with sex but with living alone. Given the fact that students living alone tend to live closer to the university, indicating that they need less time to commute to school, it was expected, to some extent, that they would be able to sleep longer than the participants living with their families. Previous studies among adult workers reported that long commute times were associated with self-reports of short sleep duration [23] and actigraphy-measured short sleep duration and regularity [24]. Thus, future studies should conduct in-depth analyses of the relation between commute time to university and students living with family. Although there were some differences in students' sleep duration by grade, the trends were not the same during weekdays and weekends. The students' curricula by grade may be related to these differences, and further studies are necessary.

This study has some limitations. First, our sample was limited to participants recruited from a single medical university in Japan. Therefore, the results may not be generalizable. However, we do have data from two years before the COVID-19 pandemic and could hence continuously follow the participants. Second, because we could not conduct a written survey in 2020, an online survey was conducted a couple of months later than planned, and the response rate decreased. Therefore, the risk of non-response bias due to potential differences between the responders and the non-responders should be considered when interpreting the results. Students who responded to online survey in 2020 might have had higher accessibility to the internet, more interest in surveys and sleeping habits, and more screen-viewing time. Meanwhile, the participants answered the questionnaires in their private spaces in 2020, while they did so in public spaces in 2018 and 2019. Third, because the survey in 2020 was conducted at a different time than the surveys in 2018 and 2019, it is possible that seasonal differences, such as changes in sunlight exposure time and the new school term, may have impacted the differences in sleeping habits between 2018–2019 and 2020. Fourth, our data were based on subjective answers, and many responses may not be true. Participants may have been reluctant to answer truthfully in the questionnaire because they had to write their names on them, and they were not anonymized. We could not collect objective data using an actigraph or portable electroencephalogram for such a large group of students. Although there were many erroneous answers and the collection rate decreased in 2020, the number of students with a delayed sleep phase increased. Furthermore, because responders were generally considered more ernest than non-responders, this result might be undervalued. It would be of benefit for students to overcome sleep deprivation, and a delayed sleep phase has a negative influence on students' life and health [25]. Finally, because we did not divide the participants into groups with and without remote teaching, we cannot show the relation between remote teaching and sleep duration directly. A recent study by the University of Tsukuba, Japan that screened for depression reported that mental health problems had doubled [26], and there may be other factors that influence students' sleeping habits, such as decreased physical activity, length of screen-viewing time, length of sunlight exposure time, psychological problems including depression, and the amount of alcohol consumption. Despite the above limitations, the participants' sleeping habits were examined longitudinally through this annual survey.

Although active learning has been recommended for medical education, the COVID-19 pandemic forced a shift to remote teaching so that education could continue under social restrictions. Remote teaching does not only have negative influences as described above. It can also positively help by saving school commute time. We strongly recommend the sole use of live streams or restricting assess to on demand lecture videos during the middle of the night and the use of interactive lessons on the web, to preserve the sleep-awake cycle and improve remote teaching.

## Conclusions

Students' sleep duration was longer during weekdays and shortened during weekends in 2020. This difference may have resulted from remote teaching during the COVID-19 pandemic, which also might be related to the sleep–wake phase being delayed in some students. Although it is unknown if these changes are reversible, remote teaching should be improved in terms of reducing its effect on the sleep–wake cycle. Furthermore, a follow-up review should be conducted in the next few years to examine these aspects.

## Data Availability

It is not possible to share the raw research data publicly because data privacy could be compromised. However, the raw data are available from the corresponding author. Any researcher interested in gaining access to the raw data can send their request to the corresponding author at the contact information mentioned in the manuscript. All data generated or analyzed during this study are included in this published article.

## Abbreviation

COVID-19: Coronavirus disease 2019
